# Tick saliva protein fraction inhibits breast cancer by decreasing cell viability and promoting apoptosis *in vitro*

**DOI:** 10.1371/journal.pone.0331779

**Published:** 2025-09-17

**Authors:** Ana Carolina Prado Sousa, Mario Durán-Prado, Margarita Villar, Almudena González-García, Matias Pablo Juan Szabó, José de la Fuente

**Affiliations:** 1 Ixodologia Laboratory, Faculty of Veterinary Medicine, Federal University of Uberlandia, Campus Umuarama, Uberlândia, Minas Gerais, Brazil; 2 Oxidative Stress and Neurodegeneration Group, Medical Sciences Department, Medical School, UCLM, Regional Centre for Biomedical Research, Research Institute of Castilla-La Mancha (IDISCAM), University of Castilla-La Mancha, Ciudad Real, Spain; 3 Department of Medical Sciences, School of Medicine at Ciudad Real, University of Castilla-La Mancha, Ciudad Real, Spain; 4 SaBio. Instituto de Investigación en Recursos Cinegéticos IREC-CSIC-UCLM-JCCM, Ciudad Real, Spain; 5 Biochemistry Section, Department of Inorganic, Organic Chemistry and Biochemistry, Faculty of Sciences and Chemical Technologies, Universidad de Castilla-La Mancha, Ciudad Real, Spain; 6 Department of Veterinary Pathobiology, Center for Veterinary Health Sciences, Oklahoma State University, Stillwater, Oklahoma, United States of America; University of Massachusetts Amherst, UNITED STATES OF AMERICA

## Abstract

Tick saliva contains protein and non-protein bioactive molecules with potential therapeutic applications, including anticancer properties. In this study, the effects of protein and non-protein fractions of saliva from different tick species (*Amblyomma* spp. and *Rhipicephalus sanguineus*) at various concentrations (0.1–10 µg/mL) were tested on the viability, apoptosis, and necrosis of epithelial MDA-MB-231 breast cancer cells and spontaneously immortalized HaCaT cell control. Chemical composition of tick saliva from *A. parvum* and *A. sculptum* was characterized by proteomics analysis. Cell viability was assessed using a calcein-based fluorescence method. Hoechst staining quantified the total number of cells per well, while apoptosis and necrosis were evaluated using Hoescht and propidium iodide assays, respectively. On MDA-MB-231 cancer cells, the protein fraction from *A. parvum* and *R. sanguineus* significantly reduced cell viability at the highest concentration (10 µg/mL), whereas *A. sculptum* and non-protein fractions showed no significant effect. Hoechst staining revealed a significant reduction in cell attachment at 10 µg/mL for *A. parvum* and *R. sanguineus*. A modest apoptosis (3–8%) was induced by the protein fractions at concentrations as low as 0.1 µg/mL for *R. sanguineus* and 10 µg/mL for the non-protein fraction of *A. sculptum*. Necrosis was not observed, except for a slight increase (1%) with the protein fraction of *R. sanguineus* at 10 µg/mL. No effect of *A. americanum* tick saliva protein and non-protein fractions was observed in HaCaT control cells. The results of highly represented proteins identified in *Amblyomma* spp. correlated with effect of tick saliva protein fraction on cancer cells, highlighting the potential anticancer properties of tick saliva protein fraction, which can induce apoptosis and inhibit cell attachment in breast cancer cells. These findings provide a basis for future studies of tick saliva components as novel therapeutic agents and identifying active biomolecules and mechanisms of action.

## Introduction

The search for novel therapeutic agents derived from natural sources has increased over the past decade, with particular focus on bioactive molecules from hematophagous arthropods such as ticks [1]. Ticks have evolved complex salivary secretions that enable successful blood feeding by modulating host immune responses, coagulation, and tissue repair mechanisms [2,3]. This unique salivary composition represents a rich source of molecules with potential applications in biomedicine, including anti-inflammatory, anticoagulant, and anticancer activities [4–6]. Additionally, carbohydrate modifications with α-gal epitope (Galα1–3Galβ1–4GlcNAc-R) in tick saliva proteins and lipids [7] have the potential application for therapy of primary tumors [8]. While much research has focused on the immunomodulatory properties of tick saliva, its potential as a source of anticancer agents remains underexplored.

Breast cancer is the most common cancer among women and the second cause of death from cancer among women worldwide [9]. The TNBC is characterized by the absence of estrogen receptor, progesterone receptor, and human epidermal growth factor receptor 2 (HER2), resulting in a highly aggressive phenotype that lacks targeted therapeutic options and is associated with poor prognosis [10]. Conventional treatments, including chemotherapy, often yield suboptimal outcomes, necessitating the urgent development of alternative therapeutic strategies [11]. Given the critical need for novel treatments, exploring unconventional sources of bioactive molecules, such as tick saliva, has emerged as a promising area in oncology [12].

This study aims to investigate the cytotoxic and pro-apoptotic effects of protein and non-protein fractions of salivary secretions from *Amblyomma parvum*, *Rhipicephalus sanguineus* sensu lato, and *Amblyomma sculptum* on MDA-MB-231 cells, a model for TNBC research. The objective is to identify and characterize novel bioactive molecules that may serve as therapeutics for TNBC, contributing to the diversification of treatment strategies for this challenging disease. The chosen tick species are known to bite humans and thus with a saliva with potential to modulate human host´s responses [13,14]. Among these tick adults, *A. parvum* were shown to successfully parasitize an array of hosts indicating a pluripotential saliva [15].

The relevance of this research lies in its potential to expand the repertoire of anticancer agents through the identification of unique bioactive compounds from tick saliva. This approach leverages the evolutionary adaptations of ticks, which have developed salivary proteins capable of modulating cellular pathways that may be repurposed to inhibit cancer progression [16]. By elucidating the anticancer properties of these molecules, this study aims to address the pressing need for innovative therapeutic approaches in TNBC management.

The study contributions extend beyond cancer research, offering insights into the broader applicability of tick-derived biomolecules in other areas of biomedical sciences. Additionally, understanding the molecular interactions between tick salivary components and cancer cells may contribute to future investigations into the role of these molecules in immune modulation and infectious disease control. This interdisciplinary approach underscores the value of natural bioactive compounds in advancing translational research and therapeutic development.

## Materials and methods

### Ethics statement

All animal procedures were conducted in compliance with the ethical standards and regulations of the Brazilian National Council for the Control of Animal Experimentation (CONCEA). The study protocols were approved by the Ethics Committee on Animal Use of the Federal University of Uberlândia (CEUA/UFU), under protocol numbers 033/14 and 069/18. During the experiment, rabbits were housed individually in clean, tall cages that allowed them to stand upright on their hind limbs, in accordance with recommended welfare standards. The animals were kept in a controlled animal facility with regulated temperature and humidity. They had ad libitum access to filtered water and standard commercial feed, and cages were cleaned daily to ensure proper hygiene and animal welfare. Tick infestations were performed within feeding chambers non-invasively affixed to the animals’ dorsal region using a non-toxic adhesive. Mild local inflammation was observed at tick attachment sites, but these effects were generally mitigated by the anti-inflammatory, analgesic, and anti-pruritic properties of the tick saliva. At the end of the study, rabbits were humanely euthanized by deep anesthesia followed by barbiturate overdose, in accordance with institutional and national guidelines. For *A. americanum* tick feeding, cattle were housed with the approval and supervision of the Texas A&M University Institutional Animal Care and Use Committee, USA (2020-0094) [7].

### Ticks

Laboratory tick colonies of *A. sculptum*, *A. parvum* and *R. sanguineus* were kept in the Laboratory of Ixodology of the Faculty of Veterinary Medicine, Federal University of Uberlandia (LABIX-UFU). The ticks were fed inside feeding chambers glued to the shaved back of New Zealand white rabbits [17]. Unfed ticks of each species were released inside the feeding chamber for both colony maintenance and for saliva collection. Unfed ticks and those that had already fed were kept in a humid incubator at 27 °C and 80% humidity. The *A. americanum* tick colony was maintained and saliva collected under similar conditions at Texas A&M University [7].

### Tick saliva collection

For saliva collection, unfed adult pathogen-free ticks, 25 females and 10 males per chamber, were used (Table 1). Rabbits and cattle were always tick-bite naïve and different hosts were used for each tick species (two chambers per rabbit with four rabbits per tick species). Partially engorged and engorged females were collected, usually 7–10 days after released into the feeding chamber, and cleaned with 10% PBS at room temperature (25 ºC). The ticks were then fixed to double-sided tape on a flat, rigid surface and cleaned with 70% alcohol. To stimulate salivation,10–20 µl of a 0.2% Dopamine solution was inoculated into the hemocele of each tick, using a 1.0 syringe and a 12.5 x 0.33 mm needle. Secreted saliva was collected with an automatic pipette, stored in 2.0 mL microtubes and kept in ice during the collection period. Afterwards the saliva was filtered (22 µm pores) and stored at 80 ºC until use. Several collections were carried and the quantity used for lyophilization was derived from a pool of different collections (Table 1).

**Table 1:** Amount of saliva collected per species and number of ticks.

Tick species (weight of engorged females)*	Number of ticks	Amount of saliva
*Amblyomma sculptum* (490–840 mg)	10	250 µL
*Amblyomma parvum* (300–500 mg)	35	250 µL
*Amblyomma americanum* (210–630 mg)	30	250 µL
*Rhipicephalus sanguineus* (140–210 mg)**	100	250 µL

*References for *A. sculptum* [18], *A. parvum* [15] and *R. sanguineus* [19]. **Tropical lineage (*Rhipicephalus linnaei*).

### Proteomics analysis of tick saliva

For a preliminary analysis of tick salivary proteome, the proteomics was conducted in *A. sculptum* and *A. parvum* species, but not in *R. sanguineus* because lower weight of engorged females and thus the high number of ticks required to collect enough saliva for analysis.

### Protein digestion with trypsin

After protein quantification in tick saliva by spectrophotometric analysis with a BCA method using a commercial kit and bovine serum albumin (BSA) for the standard curve (G-Biosciences, St. Louis, MO, USA), 100 µg of proteins were subjected to trypsin digestion. To enhance the enzymatic digestion efficiency, the surfactant RapiGest SF (Waters, Milford, MA, USA) was used. The sample was first reduced with Dithiothreitol (Sigma-Aldrich, St. Louis, MO, USA) and then alkylated with Iodoacetamide (Sigma-Aldrich). Subsequently, trypsin digestion was performed using Promega (Madison, WI, USA) trypsin enzyme at 37°C for 16 hours. The enzymatic activity was halted by adding a 0.5% trifluoroacetic acid (TFA) solution (Sigma-Aldrich). The resulting peptides were purified using a C18 tip purification method (Bond Elut OMIX, Agilent Technologies, Santa Clara, CA, USA). After drying in a sample miVac Duo concentrator (Genevac Ltd., SP Industriees, Inc., Ipswich, UK), the sample was solubilized in 0.1% TFA and transferred to a specific vial for proteomics analysis.

### Mass spectrometry analysis

The mass spectrometry analyses were performed using an Agilent Infinity 1260 liquid chromatograph (Agilent) with chromatographic column AdvanceBio Peptide Mapping model (Agilent, internal diameter 2.1 mm, length 10 cm, 2.7 μm particle size) coupled to the high-resolution mass spectrometer with an electrospray ionization source (6520B Q-TOF, Agilent) at the Nanobiotechnology Laboratory of the Federal University of Uberlândia, Brazil.

The chromatographic parameters involved the use of (A) water and (B) acetonitrile as the mobile phase, both acidified with formic acid (0.1% v/v). The gradient used was of 2% B (0 min), 2% B (10 min), 15% B (40 min), 50% B (150 min), 70% B (200 min), 98% B (220 min), 98% B (300 min), 100% B (301 min), and 100% B (400 min), at a flow rate of 400 µL/min. The ionization parameters employed a nebulizer pressure of 45 psi, a drying gas flow rate of 8 L/min at a temperature of 325 °C, and an energy of 4 kV applied to the capillary.

### Protein identification and analysis

Protein identification was carried out by considering the high-resolution mass with an error of less than 10 ppm and the mass-to-charge ratio spectra (m/z). These data were compared with a database. The analysis was conducted using the Spectrum Mill MS Proteomics Workbench program, developed by Agilent Technologies. The NCBI database (https://www.ncbi.nlm.nih.gov) was used for the protein identification process.

### Protein functional annotations and classifications

BLASTP searches were conducted against multiple databases to annotate the matched proteins. For tick protein identification, the following databases were used: non-redundant (NR), Acari, and refseq-invertebrate from NCBI, Acari from UniProt, the Gene Ontology (GO) FASTA subset, MEROPS database, and the conserved domains database of NCBI containing COG, PFAM, and SMART motifs. To annotate rabbit proteins to discard, the following databases were used: *Oryctolagus cuniculus* and refseq-vertebrates from NCBI, *O. cuniculus* from UniProt, the Gene Ontology (GO) FASTA subset, and the conserved domains database of NCBI containing COG, PFAM, and SMART motifs.

### Tick saliva lyophilization

The tick saliva protein and non-protein fractions were subjected to the lyophilization. First, the tick saliva samples were placed in 2 mL microtubes, which were capped and sealed with partially perforated parafilm, and then immersed in liquid nitrogen for 5 minutes. Next, the samples were placed in a lyophilizer, which was calibrated with temperature parameters set to −40 °C and vacuum pressure at approximately 500 µHg for 20 hrs. The tick saliva samples were then removed from the equipment, labeled, sealed and stored at −20 °C until use.

### Tick saliva protein and non‑protein fractions

The methodological approach used to prepare tick saliva protein and non-protein fractions was previously validated [7,20]. Tick saliva (250 µL, 20 µL for *A. americanum*) was diluted 1:1 in PBS and filtered twice through an Amicon 10 kDa unit (Merck & Co., Inc., Kenilworth, NJ, USA). Of this, 80% passed through the Amicon membrane and was considered the non-protein fraction. The fraction that did not pass through the Amicon membrane was considered the protein fraction. Protein concentration was assessed spectrophotometrically with a BCA method using a commercial kit and BSA for the standard curve (G-Biosciences).

### Assessment of cell viability, apoptosis and necrosis

The MDA-MB-231 breast cancer cells epithelial cells (ATCC HTB-26) and HaCaT cells spontaneously transformed aneuploid immortal keratinocyte cell line derived from adult human skin were grown in Eagle’s minimal essential medium (EMEM, Sigma-Aldrich), supplemented with inactivated 10% fetal calf serum, 2 mM L-glutamine, 1 mM sodium pyruvate, 0.1 mg/mL streptomycin and 0.1 mg/mL ampicillin, maintained at 37 °C in 5% CO_2_. For cells viability and related assays, cells were cultured in optical 96-well plates at a density of 20x10^5^ (1-2x10^4^) cells/well. Protein fraction of tick saliva was added to the cultures to reach the final concentrations of 0.1, 1 and 10 µg/mL, testing in parallel an equivalent volume of the non-protein fraction and PBS as control. After 72 h incubation, cells were loaded with 1 µM calcein-AM (Thermo Fisher Scientific Inc., Waltham, MA, USA), 1 µM propidium iodide (Sigma) and 1 µM Hoechst (Thermo Fisher) for 30 min to assess viability, necrosis and apoptosis as described previously [21,22]. Whole-well fluorescence for green, red and blue channels was recorded by microfluorimetry and well were scanned for each channel with a 20x objective using a Cytation 5 equipment (BioTek, Agilent, Santa Clara, CA, USA). Blue fluorescence indicates the total number of cells. Cell viability was calculated from green fluorescence, normalizing to blue fluorescence. Necrosis was calculated with Fiji-ImageJ (https://imagej.net/software/fiji/) by counting nuclei stained with propidium iodide, referred to the total number of cells, quantified from Hoechst 33342 fluorometric assay (BMG LabTech, Ortenberg, Germany). Results are expressed as percentage of necrotic *vs.* total cells. Apoptosis was assessed by qualitative image-based analysis as previously reported and validated [21]. This technique identifies highly stained nuclei (indicative of early apoptosis) before nuclear envelope vesiculation occurs (late apoptosis), offering a straightforward and robust approach. Early apoptosis was considered as isolated small and condensed nucleus and late apoptosis as vesiculated nuclei. Results are expressed as percentage of apoptotic cells *vs.* total cells.

### Statistical analysis

Data were expressed as mean ± standard error or the mean (S.E.M.), obtained from three independent experiments. Statistical analysis was carried out in comparison to PBS-treated controls with GraphPad Prism 8, using one-way ANOVA (Bonferroni test), p < 0.05 (n = 3 replicates, n = 2 replicates for HaCaT control cells).

## Results

Different volume of saliva was obtained per tick species in accordance with the weight of engorged females (Table 1). Accordingly, differences in saliva secreted between tick species are probably associated with engorged female weight (e.g., [23]).

The characterization of tick saliva proteome in *A. sculptum* and *A. parvum* species provided a preliminary analysis of proteins with possible functional implications in anticancer properties (S1 and S2 Tables, S1 and S2 Figs in S1 Supporting Information). In *A. parvum*, the largest number of identified proteins belong to the protease inhibitor family (15.6%) while in *A. sculptum* cyclophilin-type PPIASE family was the most represented (11.1%).

For this study, a calcein-based method was chosen as the primary approach to assess the effect of protein and non-protein fractions from tick saliva on MDA-MB-231 breast cancer cells. This technique provides several advantages that justify its selection as a reliable and informative method for initial viability screening [21,22]. The presence of fluorescence correlates directly with the metabolic activity and viability of the cells. This method is widely recognized for its sensitivity, reproducibility, and ability to provide quantitative data on live cells without damaging them, making it ideal for assessing subtle cytotoxic effects. This approach not only facilitates the identification of candidate bioactive compounds but also lays the foundation for further investigations into the molecular mechanisms underlying the observed effects.

**Fig 1:**
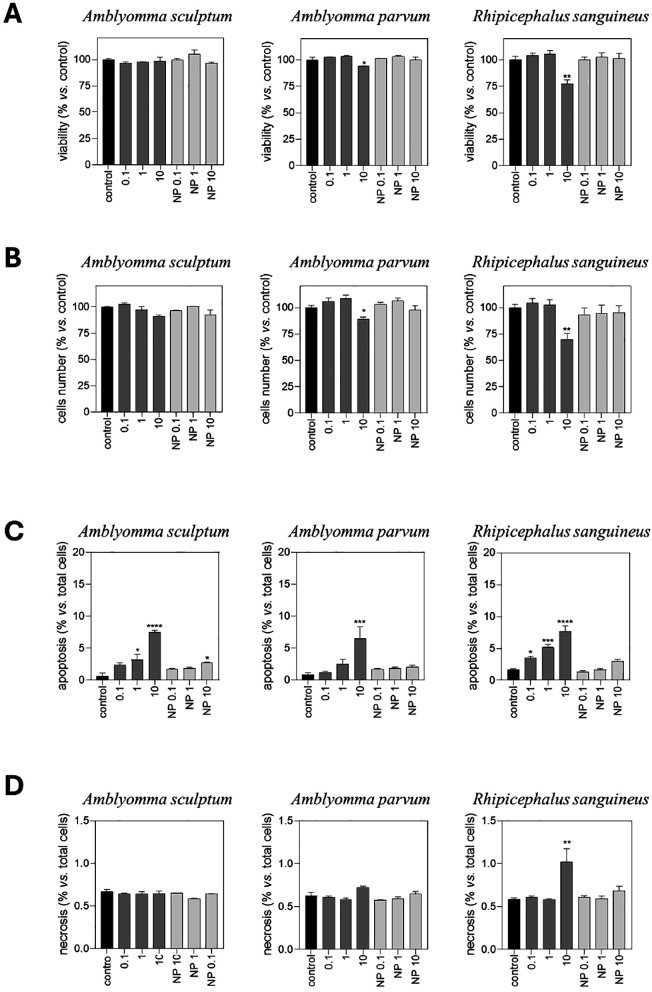
Effect of tick saliva on MDA-MB-231 cells. **(A)** Viability of MDA-MB-231 cells exposed to tick saliva. Protein and non-protein (NP) fractions of saliva from *A. sculptum*, *A. parvum* and *R. sanguineus* were diluted in DMEM at the indicated concentrations and then added to subconfluent MDA-MB-231 cells. After 72 hrs of incubation, cells were loaded with calcein-AM to stain viable cells. Viability was quantified by microfluorimetry. **(B)** Tick saliva affects the number of attached MDA-MB-231 cells. Protein and non-protein fractions of saliva from *A. sculptum*, *A. parvum* and *R. sanguineus* were diluted in DMEM at the indicated concentrations and then added to subconfluent MDA-MB-231 cells. After 72 hrs of incubation, cells were loaded with Hoescht to stain cell nuclei. Total nuclei were quantified by microfluorimetry. **(C)** Protein fraction of tick saliva induced apoptosis in MDA-MB-231 cells. Protein and non-protein fractions of saliva from *A. sculptum*, *A. parvum* and *R. sanguineus* were diluted in DMEM at the indicated concentrations and then added to subconfluent MDA-MB-231 cells. After 72 hrs of incubation, cells were loaded with Hoescht to stain cell nuclei. Apoptosis was evaluated by morphological criteria, considering positive small and condensed nuclei as well as lobulated nuclei and is presented as percentage of apoptotic cells vs. total cells. **(D)** Effect of tick saliva on necrosis of MDA-MB-231 cells. Protein and non-protein fractions of saliva from *A. sculptum, A. parvum* and *R. sanguineus* was diluted in DMEM at the indicated concentrations and then added to subconfluent MDA-MB-231 cells. After 72 hrs of incubation, cells were loaded with propidium iodide to stain cell nuclei of necrotic cells. Necrosis was evaluated by microfluorimetry and represented as percentage of necrotic cells vs. total cells. Results show the average of n = 3 replicates, and the error is shown as **S.**E.M. One-way ANOVA Bonferroni test vs. PBS control, ****p < 0.0001; ***p < 0.001; **p < 0.01; *p < 0.05.

**Fig 2:**
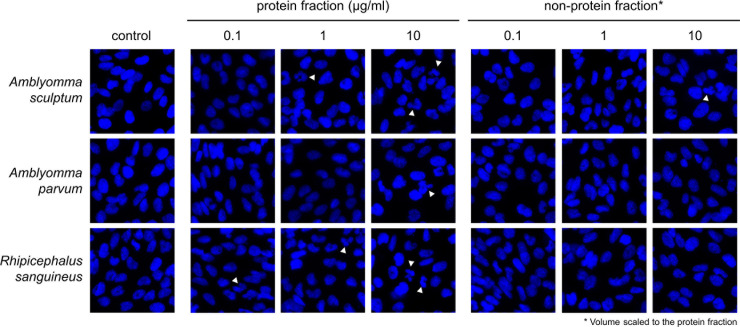
Apoptosis of MDA-MB-231 cells exposed to tick saliva. Protein and non-protein fractions of tick saliva were diluted in DMEM at the indicated concentrations and then added to subconfluent MDA-MB-231 cells. PBS-treated cells were used as control. After 72h of incubation, cells were loaded with Hoechst to stain nuclei. Apoptosis was evaluated by morphological criteria, considering positive small and condensed nuclei as well as lobulated nuclei (white arrows).

The results showed an inhibitory effect by reducing cell viability for the highest concentration (10 µg/mL) of the protein fraction of saliva from *A. parvum* and *R. sanguineus*, and no significant effect of *A. sculptum* saliva (Fig. 1A). In second term, the total number of cells per well was determined by quantifying Hoechst-stained nuclei (Fig. 1B). This approach revealed a decrease in the total number of cells attached to the culture plate at the highest concentration (10 µg/mL) of the protein fraction of saliva from the three tick species, being significant only for *A. parvum* and *R. sanguineus.* No effect was observed for any NP fraction (Figs. 1A and 1B). The next step was to evaluate if any of the fractions was affecting cell apoptosis (Figs. 1C and 2). Protein fractions from the three species were able to elicit apoptosis even at very low protein concentrations of 0.1 µg/mL for *R. sanguineus,* being also significant for the highest amount of NP fraction of *A. sculptum* saliva. However, though significant, apoptosis ranged between 3% and 8% (Figs. 1C and 2).

Finally, potential necrotic effect of tick saliva was determined (Fig. 1D). Results indicated no pro-necrotic effect of tick saliva, except for the highest concentration of the protein fraction of *R. sanguineus* that raises necrosis to 1% *vs.* total cells.

**Fig 3:**
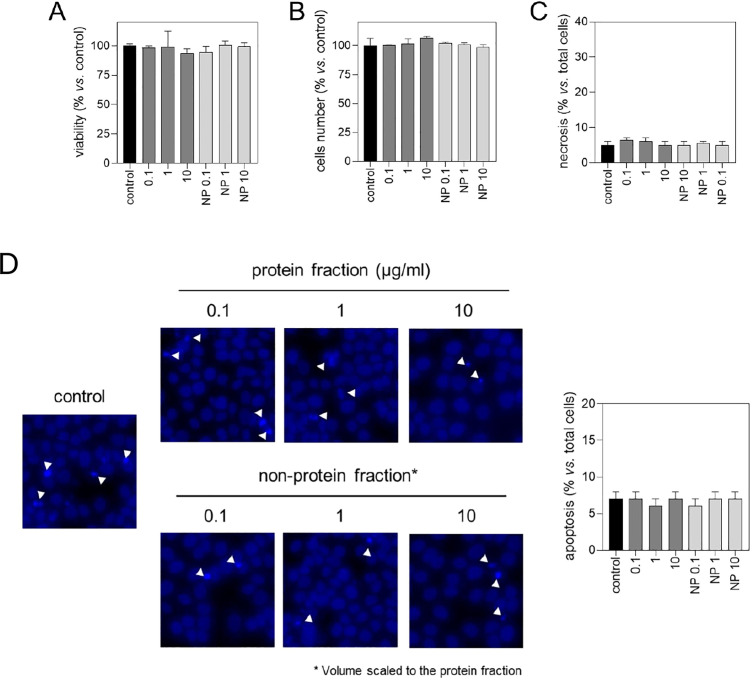
Effect of tick saliva on HaCaT cells. **(A)** Viability of HaCaT cells exposed to tick saliva. Protein and non-protein (NP) fractions of saliva from *A. americanum* were diluted in DMEM at the indicated concentrations and then added to subconfluent HaCaT cells. After 72 hrs of incubation, cells were loaded with calcein-AM to stain viable cells. Viability was quantified by microfluorimetry. **(B)** Attached HaCaT cells. Protein and non-protein fractions of saliva from *A. americanum* were diluted in DMEM at the indicated concentrations and then added to subconfluent HaCaT cells. After 72 hrs of incubation, cells were loaded with Hoescht to stain cell nuclei. Total nuclei were quantified by microfluorimetry. **(C)** Effect of tick saliva on necrosis. Protein and non-protein fractions of saliva from *A. americanum* was diluted in DMEM at the indicated concentrations and then added to subconfluent HaCaT cells. After 72 hrs of incubation, cells were loaded with propidium iodide to stain cell nuclei of necrotic cells. Necrosis was evaluated by microfluorimetry and represented as percentage of necrotic cells vs. total cells. **(D)** Effect of tick saliva on apoptosis. Protein and non-protein fractions of tick saliva were diluted in DMEM at the indicated concentrations and then added to subconfluent HaCaT cells. PBS-treated cells were used as control. After 72h of incubation, cells were loaded with Hoechst to stain nuclei. Apoptosis was evaluated by morphological criteria, considering positive small and condensed nuclei as well as lobulated nuclei (white arrows). Results are represented as percentage of apoptotic cells vs. total cells.

As control, human HaCaT non-cancerous spontaneously transformed aneuploid immortal keratinocyte cells were treated with *A. americanum* tick saliva protein fractions and results showed no effect, thus supporting effect of tick biomolecules only in cancer epithelial cells (Figs. 3A-3D).

## Discussion

This study addressed the effect of protein and non-protein fractions from tick saliva on the viability, proliferation, apoptosis, and necrosis of MDA-MB-231 breast cancer cells and HaCaT non-cancerous spontaneously transformed aneuploid immortal keratinocyte cells, with the aim to contribute to the understanding of how tick saliva compounds might influence cancer cells. Our findings revealed several key insights as well as limitations that require further investigation to fully elucidate the mechanisms in response to tick salivary fractions.

Currently, breast cancer is one of the major cause of death among women globally [9]. In developed nations, it ranks as the second leading cause of cancer-related mortality, following lung cancer [24]. Typically, breast cancer treatment involves surgery, such as unilateral or bilateral mastectomy, which can be either partial or complete depending on the tumor’s location and size. A significant amount of research has focused on finding less invasive and less damaging breast cancer treatments using therapeutic biomolecules from plants and animals. Tick saliva, a secretion containing a diverse range of bioactive molecules [25], has been investigated for its potential anti-cancer properties [26–28].

The collection of 250 microliters of tick saliva required the use of different numbers of individual ticks for each species, reflecting the considerable investment in biological resources required for this study. Each tick typically produces only small volumes of saliva per extraction session, highlighting the logistical challenges associated with acquiring enough for experimental use. It is important to note that the *R. sanguineus* colony used here pertains to the tropical lineage of the *R. sanguineus* complex and the designation *Rhipicephalus linnaei* has been proposed for this lineage [29].

The first experimental approach evaluated cell viability using a calcein-based assay. The results demonstrated an inhibitory effect on MDA-MB-231 cell viability when exposed to the protein fraction from *A. parvum* and *R. sanguineus* at a concentration of 10 µg/ml, with no significant effect observed for the protein fraction from *A. sculptum*. These findings are in line with previous studies suggesting that tick saliva proteins can exhibit bioactive properties with potential cytotoxic effects on cancer cells. For instance, protein fractions from other tick species have been shown to impact cell viability by modulating host immune responses or directly affecting cellular signaling pathways [30,31]. The lack of an effect by *A. sculptum* could be related to the specific protein composition of the saliva from this species.

Furthermore, quantification of Hoechst-stained nuclei revealed that the total number of cells attached to the culture plate decreased significantly at the highest protein concentration of *A. parvum* and *R. sanguineus*, but not for *A. sculptum*. These results support the idea that protein fractions from *R. sanguineus* and *A. parvum* may influence cell adhesion or growth, possibly via interaction with cell surface receptors or changes in the extracellular matrix, both of which are known to affect tumor cell proliferation [32].

In not tumor HaCaT control cells tick salivary fractions did not show an effect. Sousa et al. [5] evaluated the effect of saliva of the same tick species on MCF-7, MDA-MB-231 breast cancer cell lines and on the non-neoplastic MCF-10A cell line. They observed that tick saliva from all three tick species showed cytotoxicity to tumor cells (MCF-7, MDA-MB-231) but not to the non-tumor cells (MCF-10A). All three types of tick saliva used in their study caused cell rounding, reductions in cell count and alterations in cell size and arrangement. MCF-7 underwent the greatest changes relative to MDA-MB-231 and MCF-10A. Nevertheless, MDA-MB-231 showed several abnormalities, mainly cell rounding and lower cell counts [5]. Akagi et al. [27] observed the pro-apoptotic effects of Amblyomin X, a Kunitz-type protease inhibitor derived from *A. sculptum* tick saliva, on murine renal cancer cells. The researchers reported morphological changes in cancer cells that are indicative of apoptosis, such as shrinkage, cytoskeletal rearrangement, and DNA fragmentation. The mechanism by which Amblyomin X affects tumor cells was mediated by increase in the expression of dynein subunits, which are involved in intracellular transport and enhanced both gene and protein expression of various subunits of the molecular motor dynein, which is crucial for intracellular transport. [31]. In this study, Amblyomin-X was specifically internalized by tumor cells via lipid-raft endocytic pathways, but not by fibroblasts. Additionally, the dynein inhibitor ciliobrevin A reduced Amblyomin-X uptake in tumor cells, and when tumor cells were incubated with this protein, the trypsin-like activity of the proteasome was inhibited, an effect that was reversed by pretreatment with ciliobrevin A. The study also highlighted the protein’s specific uptake by tumor cells via lipid-raft endocytic pathways and its ability to inhibit the proteasome activity in these cells, leading to pro-apoptotic effects [31].

Štibrániová et al. [26] demonstrated that tick saliva, specifically from *Ixodes ricinus*, contained bioactive molecules that could affect tumor cell lines. The authors discussed the immunomodulatory and anti-tumor properties of these proteins, particularly their potential to alter the immune response in the tumor microenvironment [26]. The evaluation of the potential anticancer effects of salivary proteins from *Ixodes scapularis* showed how these proteins modulate the immune response and interfere with tumor cell proliferation, but the mechanisms were not fully elucidated [28].

Interestingly, the NP fraction of saliva of all three tick species did not significantly affect cell viability or proliferation, suggesting that protein components are likely the primary contributors to the observed effects on MDA-MB-231 cells. Nevertheless, studies suggest that the non-protein fraction may also play a significant role in modulating host immune responses and influencing cellular activities. A study by Oliveira et al. [33] demonstrated that non-protein fractions from *R. sanguineus* saliva could alter the host’s immune responses by modulating cytokine production and inhibiting inflammatory pathways. These effects were not solely attributed to proteins but also to smaller bioactive molecules present in the non-protein fraction, such as lipids and metabolites, which have been shown to influence the host’s immune system, thereby promoting tick survival and facilitating infection transmission.

We also observed that the protein fractions from all three tick species could elicit apoptosis in MDA-MB-231 cells, even at low concentrations (e.g., 0.1 µg/mL for *R. sanguineus*). This finding is consistent with previous studies highlighting the apoptotic potential of tick saliva proteins [34]. While apoptosis was significant for the highest concentration of the non-protein fraction from *A. sculptum*, it was minimal (3–8%) across the tested concentrations, suggesting that the observed apoptosis was likely a result of direct or indirect effects of the protein fractions. The apoptotic potential of these saliva proteins is a novel and relevant aspect of this study, as induction of programmed cell death in cancer cells is a highly desirable therapeutic outcome. However, it is important to consider the relatively low percentage of apoptosis observed (3–8%), as this suggests that while the tick saliva proteins may have apoptotic activity, their efficiency in triggering this response in MDA-MB-231 cells is limited. This could be attributed to factors such as the concentration-dependent response or the need for higher doses to achieve a more potent apoptotic effect. The study by Kazimirová et al. [24], showed an apoptotic and dose-dependent inhibitory effects of salivary gland extracts of four hard tick species on the proliferation of HeLa cells, suggesting that salivary gland extract (SGE) of various hard tick species may contain different compounds that suppress cell proliferation and induce apoptosis, and the presence and amounts of the active compounds change during tick feeding.

Amblyomin-X has been extensively studied in pre-clinical trials and is currently being developed as a potential treatment for cancer [35]. It specifically induces apoptosis in tumor cells and has been shown to reduce tumor size in vivo in melanoma animal models, as well as decrease metastasis and tumor growth in experimental studies [36]. In pre-clinical evaluations, this protein was found to significantly reduce lung metastasis in a mouse orthotopic kidney tumor model [37]. Notably, Amblyomin-X does not appear to cause any mortality, with toxicity symptoms being mild, reversible, and observed only at higher doses, indicating a favorable safety profile for injection in mice. More recent studies in horse melanomas treated with this protein demonstrated a significant reduction in tumor size [38]. Proteins bound to dynein, associated with aggresome formation, inhibition of autophagy, and markers of early and recycling endosomes, were identified only in tumor cells treated with Amblyomin-X, results that provided new insights into the anti-tumor mechanism of this protein and uncover an unexpected role for cytoplasmic dynein in its uptake, intracellular trafficking, and pro-apoptotic effects [31]. Future studies may investigate the dose-response relationship to explore the molecular mechanisms underlying the modest apoptotic effects and whether any adjuvant molecules could enhance the observed responses.

Finally, necrosis was assessed, and results showed no significant pro-necrotic effects of tick saliva fractions, except for the highest concentration of the protein fraction from *R. sanguineus*, which induced a minimal increase in necrosis (1%). This finding aligns with the idea that while necrosis can occur under certain conditions, apoptosis seems to be the predominant form of cell death induced by tick saliva in MDA-MB-231 cells. Similar results were previously shown by Sousa et al. [5], who demonstrated that tumor cells show apoptosis induced by caspase-3 and caspase 7 activity, suggesting that intrinsic apoptotic pathway may be triggered by tick saliva with necrosis being less frequently observed. Additionally, the effect of *I. scapularis* tick saliva on tumor cell lines showed immunosuppressive and anti-proliferative effects, but while apoptosis and cell cycle alterations were observed, necrosis was not notably induced in tumor cells by tick saliva [27].

The largest number of proteins identified in tick saliva by proteomics analysis were annotated as protease inhibitor and cyclophilin-type PPIASE families in *A. parvum* and *A. sculptum*, respectively. Protease inhibitors represent one of the main components of the molecular biology of tick saliva responsible for the completion of tick feeding and perpetuation and thus potential targets for anti-tick vaccines [39]. Regarding cyclophilins, these proteins have a function in tick embryogenesis [40]. In support to results presented in this study for *A. parvum* tick salivary protein fraction, protease inhibitors are potential biopharmaceuticals in cancer therapy to disrupt processes like angiogenesis, metastasis and tumor invasion [41–43]. However, in accordance with no effect with *A. sculptum* tick saliva protein fraction in cancer cells, cyclophilin is considered a possible target in cancer therapy due to its main function in enhancing cell survival under stressful conditions associated with antiapoptotic proteins, transcription factors, cell migration regulatory proteins, and upregulation of signaling proteins [44,45].

In a previous study, the characterization of *A. americanum* tick saliva NP fraction lipidome identified diacylglycerol (DAG) as the glycolipid class with highest representation [7]. The DAG kinases and dysregulation of DAG effectors activity or abundance have been associated with tumor initiation, progression and metastasis [46]. These results in another *Amblyomma* sp. support the limited or no effect of NP fraction in cancer cells.

Our study presents new insights into the bioactivity of tick saliva fractions in cancer cells without effect on non-cancer cells. The ability of tick salivary proteins to induce apoptosis and inhibit the growth of breast cancer cells offers a promising direction for the development of novel anti-cancer therapies. Furthermore, the lack of significant effects from non-protein fractions suggests that proteins are the key bioactive components, which could guide future studies toward isolating and characterizing these proteins for therapeutic use. Future studies employing high-throughput proteomic and transcriptomic analyses could identify the specific molecular targets and pathways involved, providing a more comprehensive understanding of how tick saliva interacts with tumor cells. Our findings are consistent with previous research showing that tick saliva proteins contain bioactive molecules capable of modulating host cell functions. For example, several studies have identified proteins from tick saliva that exert immunomodulatory, anticoagulant, and cytotoxic effects [7,47,48]. However, the relatively low level of apoptosis observed in this study suggests that while tick salivary proteins have potential therapeutic properties, their clinical application may require further refinement or combination with other therapeutic strategies.

## Conclusions

In conclusion, the protein fractions of tick saliva from *A. parvum* and *R. sanguineus* exhibit inhibitory effects on MDA-MB-231 breast cancer cells by decreasing cell viability and promoting apoptosis. These findings highlight the potential of tick saliva proteins as a source of novel therapeutic agents for cancer treatment. As previously reported, tick saliva does not affect the viability of non-cancerous cells [5]. Nevertheless, the main limitations of the study are (a) experiments with a single cancer cell line, (b) the use of a single representative time point for analysis due to the modest nature of the observed effect and the limited availability of tick-derived samples, (c) the lack of information of the proteins involved in the observed inhibitory effect, (d) proteomics data collected only in *Amblyomma* spp., which affects comparative analysis across tick species, (e) the use of qualitative analysis for apoptosis which should be complemented with flow cytometry, Annexin V-FITC, or caspase activity assays, and (f) that experiments were conducted only *in vitro*. To address these limitations, further studies should include other cell lines to expand the applicability of these findings to other cancer subtypes with a larger number of replicates and sampling time points to improve reproducibility and to identify the tick salivary proteins involved in this process and their functional mechanisms of action, and to explore their efficacy in other cancer models. Additionally, future research should investigate the synergistic effects of tick saliva proteins with existing chemotherapeutic agents, paving the way for more effective cancer therapies.

## Supporting information

S1 Supporting Information Data of proteomics analysis of saliva from *Amblyomma* species. (PDF)
