# Supplementary material for: Tick saliva protein fraction inhibits breast cancer by decreasing cell viability and promoting apoptosis in vitro
Source: PLoS One. 2025 Sep 17;20(9):e0331779. doi: 10.1371/journal.pone.0331779 (PMC12443249; doi:10.1371/journal.pone.0331779)

Supporting information for

Tick saliva protein fraction inhibits breast cancer by decreasing cell viability and promoting apoptosis *in vitro*

Ana Carolina Prado Sousa[1,&], Mario Durán-Prado[2,3,&], Margarita Villar[4,5], Matias Pablo Juan Szabó[1], José de la Fuente[4,6,*]

[&] Ana Carolina Prado Sousa and Mario Durán-Prado contributed equally to this work.

[1]Ixodologia Laboratory, Faculty of Veterinary Medicine, Federal University of Uberlandia, campus Umuarama, bloco 6T, Uberlândia, MG, Brazil.

[2]Oxidative Stress and Neurodegeneration Group, Medical Sciences Department, Medical School, UCLM, Regional Centre for Biomedical Research, Research Institute of Castilla-La Mancha (IDISCAM), University of Castilla-La Mancha, Ciudad Real 13071, Spain.

[3]Department of Medical Sciences, School of Medicine at Ciudad Real, University of Castilla-La Mancha, Ciudad Real 13071, Spain.

[4]SaBio. Instituto de Investigación en Recursos Cinegéticos IREC-CSIC-UCLM-JCCM, Ronda de Toledo 12, 13005 Ciudad Real, Spain.

[5]Biochemistry Section, Department of Inorganic, Organic Chemistry and Biochemistry, Faculty of Sciences and Chemical Technologies, Universidad de Castilla-La Mancha, Ave. Camilo José Cela 10, 13071 Ciudad Real, Spain.

[6]Department of Veterinary Pathobiology, Center for Veterinary Health Sciences, Oklahoma State University, Stillwater, OK 74078, USA.

*Correspondence: José de la Fuente, SaBio, Instituto de Investigación en Recursos Cinegéticos (IREC-CSIC-UCLM-JCCM), Ronda de Toledo 12, 13005 Ciudad Real, Spain. Email: jose_delafuente@yahoo.com / josedejesus.fuente@uclm.es

S1 Supporting Information. Data of proteomics analysis of saliva from *Amblyomma* species. Additional information can be found at:

Sousa, A. C. P. (2022). Carrapatos e riquétsias em javaporcos (*Sus scrofa scrofa*) em uma fazenda do município de Tupaciguara-Minas Gerais e análise proteômica da saliva dos carrapatos *Amblyomma sculptum* e *Amblyomma parvum*. https://doi.org/10.14393/ufu.di.2015.500

S1 Table. Proteins identified in *Amblyomma parvum* tick saliva.

Database Accession	Protein Name	Protein family	Protein MW(Da)
XP_029826390.1	Ubiquitin carboxyl-terminalhydrolase 24	Protease - Peptidase C19 family	207601.2
XP_029846393.1	Ubiquitin carboxyl-terminalhydrolase 24 isoform X1	Protease - Peptidase C19 family	289940.3
XP_029846394.1	Ubiquitin carboxyl-terminalhydrolase 24 isoform X2	Protease - Peptidase C19 family	262101.2
XP_029846437.1	Dipeptidyl peptidase 3	Protease - Peptidase M49 family	82847.8
XP_029830549.1	Carboxypeptidase D isoform X1	Glycoprotein - Peptidase M14 family	184582.7
XP_029830550.1	Carboxypeptidase D isoform X2	Glycoprotein - Peptidase M14 family	150509.1
XP_029834419.1	Alpha-1-macroglobulin isoform X1	Protease inhibitor I39 (alpha-2-macroglobulin) family	170274.8
XP_029834423.1	Alpha-1-macroglobulin isoform X2	Protease inhibitor I39 (alpha-2-macroglobulin) family	168795.3
XP_029834426.1	Alpha-1-macroglobulin isoform X5	Protease inhibitor I39 (alpha-2-macroglobulin) family	167631.1
XP_029834427.1	Alpha-1-macroglobulin isoform X6	Protease inhibitor I39 (alpha-2-macroglobulin) family	167830.1
XP_029834428.1	Alpha-1-macroglobulin isoform X7	Protease inhibitor I39 (alpha-2-macroglobulin) family	166665.9
XP_029834429.1	Alpha-1-macroglobulin isoform X8	Protease inhibitor I39 (alpha-2-macroglobulin) family	166398.5
XP_029834430.1	Alpha-1-macroglobulin isoform X9	Protease inhibitor I39 (alpha-2-macroglobulin) family	165792.9
XP_029834431.1	Alpha-1-macroglobulin isoform X10	Protease inhibitor I39 (alpha-2-macroglobulin) family	165165.2
XP_029834424.1	Alpha-2-macroglobulin-like protein 1 isoform X3	Protease inhibitor I39 (alpha-2-macroglobulin) family	169110.5
XP_029834425.1	Alpha-2-macroglobulin-like protein 1 isoform X4	Protease inhibitor I39 (alpha-2-macroglobulin) family	168258.7
XP_029831190.1	Spectrin beta chain isoform X1	Spectrin family	277913.5
XP_029831191.1	Spectrin beta chain isoform X2	Spectrin family	275651.1
XP_029831192.1	Spectrin beta chain isoform X3	Spectrin family	274927.1
XP_029831194.1	Spectrin beta chain isoform X4	Spectrin family	273710.9
XP_029831195.1	Spectrin beta chain isoform X5	Spectrin family	272664.7
XP_029831196.1	Spectrin beta chain isoform X6	Spectrin family	270724.5
XP_029831197.1	Spectrin beta chain isoform X7	Spectrin family	268493.1
XP_029825566.1	Spermatogenesis-associated protein20 isoform X1	SPATA6 family	96605.3
XP_029825567.1	Spermatogenesis-associated protein20 isoform X2	SPATA6 family	95998.1
XP_029825569.1	Spermatogenesis-associated protein20 isoform X3	SPATA6 family	91228.6

XP_029825570.1	Spermatogenesis-associated protein20 isoform X4	SPATA6 family	78237.6	1.52e+005
XP_029825571.1	Spermatogenesis-associated protein20 isoform X5	SPATA6 family	76481.8	1.52e+005
XP_029827191.1	Armadillo repeat protein deleted in velo-cardio-facial syndrome isoform X1	Beta-catenin family	126051.3	4.10e+004
XP_029827193.1	Catenin delta-2 isoform X2	Beta-catenin family	122971.8	4.10e+004
XP_029827194.1	Catenin delta-2 isoform X3	Beta-catenin family	120965.7	4.10e+004
XP_029827195.1	Catenin delta-2 isoform X4	Beta-catenin family	121382.2	4.10e+004
XP_029826319.1	NF-kappa-B inhibitor-interactingRas-like protein 1	Small GTPase superfamily, Ras family, KappaB-Ras subfamily	22296.1	1.95e+004
XP_029826320.1	NF-kappa-B inhibitor-interactingRas-like protein 1	Small GTPase superfamily, Ras family, KappaB-Ras subfamily	22296.1	1.95e+004
XP_029826321.1	NF-kappa-B inhibitor-interactingRas-like protein 1	Small GTPase superfamily, Ras family, KappaB-Ras subfamily	22296.1	1.95e+004
XP_029826322.1	NF-kappa-B inhibitor-interactingRas-like protein 1	Small GTPase superfamily, Ras family, KappaB-Ras subfamily	22296.1	1.95e+004
XP_029850182.1	Glutamate receptor ionotropic,kainate 2 isoform X1	Glutamate-gated ion channel(TC 1.A.10.1) family	101277.7	8.23e+004
XP_029850183.1	Glutamate receptor ionotropic,kainate 3 isoform X2	Glutamate-gated ion channel(TC 1.A.10.1) family	93238.7	8.23e+004
XP_029850184.1	Glutamate receptor ionotropic,kainate 2 isoform X3	Glutamate-gated ion channel(TC 1.A.10.1) family	91509.5	8.23e+004
XP_029833725.1	Protein retinal degeneration B isoform X1	PtdIns transfer protein family, PI transfer class IIA subfamily	149096.1	6.44e+005
XP_029833726.1	Protein retinal degeneration Bisoform X2	PtdIns transfer protein family, PI transfer class IIA subfamily	146119.5	6.44e+005
XP_029833727.1	Protein retinal degeneration B isoform X3	PtdIns transfer protein family, PI transfer class IIA subfamily	142147.0	6.44e+005
XP_029829301.1	MAM and LDL-receptor class A domain-containing protein 1	Glycoprotein	787174.2	4.97e+004
XP_029830538.1	Disintegrin and metalloproteinase domain-containing protein 11-like isoform X3	Glycoprotein	126301.1	3.84e+004
XP_029846645.1	Disintegrin and metalloproteinase domain-containing protein 11	Glycoprotein	127985.0	3.84e+004
XP_029830629.1	Piezo-type mechanosensitive ion channel component 2	Glycoprotein, Phosphoprotein - PIEZO (TC 1.A.75) family	274714.1	3.92e+005
XP_029825258.1	Alpha-(1,6)-fucosyltransferaseisoform X1	Glycosyltransferase	64986.5	1.52e+005
XP_029825259.1	Alpha-(1,6)-fucosyltransferaseisoform X2	Glycosyltransferase	64332.5	1.52e+005
XP_029834042.1	fatty-acid amide hydrolase 2-A-like isoform X1	Amidase family, Lipocalin	57984.9	4.06e+005
XP_029834043.1	fatty-acid amide hydrolase 2-A-like isoform X2	Amidase family, Lipocalin	54348.7	4.06e+005
XP_029836569.1	Carnitine O-palmitoyltransferase 1, liver isoform-like isoform X1	Carnitine/choline acetyltransferase family	90997.0	1.68e+004
XP_029836570.1	Carnitine O-palmitoyltransferase 1, liver isoform-like isoform X2	Carnitine/choline acetyltransferase family	89071.7	1.68e+004

XP_029823064.1	Protein MTO1homolog,mitochondrial isoform X1	MnmG family	75357.0	1.29e+004
XP_029823065.1	Protein MTO1 homolog, mitochondrial isoform X2	MnmG family	75122.8	1.29e+004
XP_029823620.1	Transcription factor SPT20 homolog isoform X3	Developmental protein - SPT20 family	78963.7	8.44e+004
XP_029838436.1	Transcription factor SPT20 homolog isoform X1	Developmental protein - SPT20 family	78970.8	8.44e+004
XP_002433826.2	Innexin shaking-B	Pannexin family	44793.3	8.61e+004
XP_029836718.1	Low Quality Protein: histone-lysine N-methyltransferase2C	Methyltransferase	619115.9	3.10e+005
XP_002410320.1	Testicular acid phosphatase homolog	Hydrolase - Histidine acid phosphatase family	42674.8	3.33e+003
XP_029831845.1	E3 ubiquitin-protein ligase MARCH8	Transferase	28307.9	5.90e+005
XP_002405700.2	Cytochrome P450 4C1	Monooxygenase, Oxidoreductase - CytochromeP450 family	63290.4	2.81e+004
XP_029830346.1	Cyclic AMP-dependent transcription factor ATF-2	bZIP family, ATF subfamily	61452.8	1.11e+005
XP_029847563.1	DNA primase large subunit-like	Eukaryotic-type primase small subunit family	60396.3	3.84e+004
XP_029847019.1	DNA replication licensing factor mcm5 - carrapato	MCM family	83108.7	5.14e+004
XP_029829505.1	Histone-lysine N-methyltransferase SETDB1-B isoform X2	Histone-lysine methyltransferase family, Suvar3-9 subfamily	153194.1	1.56e+005
XP_002410624.1	Peptidyl-prolyl cis-trans isomerase B	Cyclophilin-type PPIase family	21526.2	5.21e+004
XP_029835037.1	Glucose dehydrogenase	Glucose-6-phosphate dehydrogenase family	66513.1	5.71e+004
XP_002404799.2	Protein D1	Reaction centerPufL/M/PsbA/D family	18195.2	1.05e+004
XP_029829185.1	THAP domain-containing protein 7-like	No family	18945.2	8.30e+004
XP_029841953.1	WD repeat and FYVE domain-containing protein 3 isoform X1	No family	396589.9	1.95e+004
XP_029841954.1	WD repeat and FYVE domain-containing protein 3 isoform X2	No family	396589.9	1.95e+004

Database accession on NCBI database (https://www.ncbi.nlm.nih.gov).

S1 Fig. **Protein families identified in *Amblyomma parvum* tick saliva**. The largest number of identified proteins belong to the protease inhibitor family (15.6%).

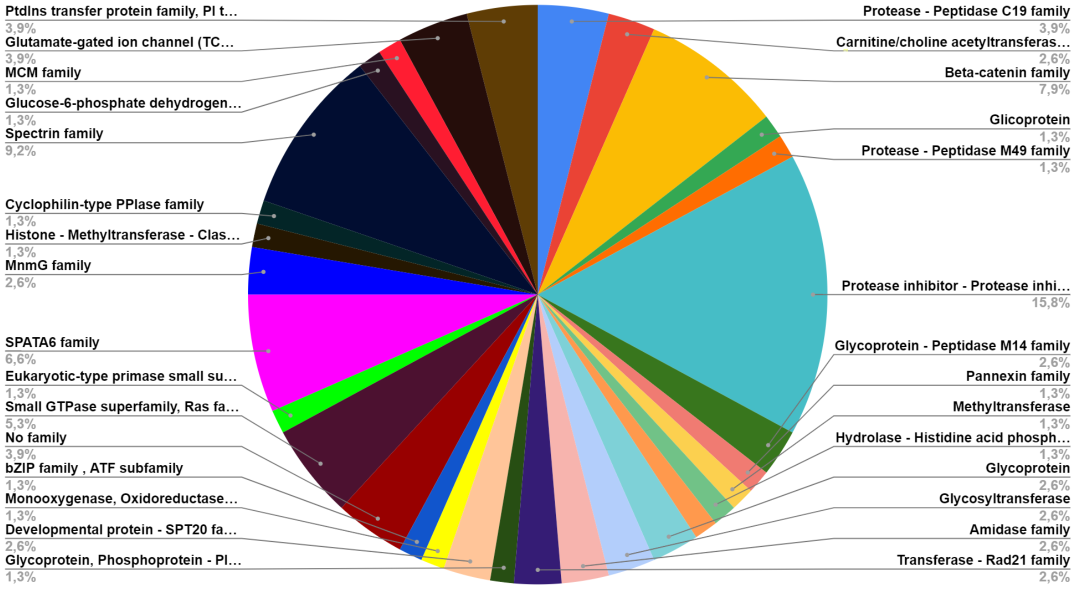

S2 Table. Proteins identified in *Amblyomma sculptum* tick saliva.

Database Accession #	Protein Name	Protein Family	Protein MW (Da)
XP_029828446.1	Cytochrome P450 3A9	Cytochrome P450	57157,7
XP_029851618.1	Condensin-2 complex D3 subunit	Phosphoprotein	177314,1
XP_029827135.1	Protein 2 expressed from heart and neural crest derivatives	Phosphoprotein	22399,9
XP_029826337.1	PRRC2A protein (isoforms X5-X11)	Phosphoprotein/Glycosyltransferase	175137,1
XP_029830823.1	Venom metalloproteinase antarease-like TtrivMP_A	Glycoprotein	63685,3
XP_029841229.1	LOW-QUALITY PROTEIN: laminin alpha-1 subunit	Glycoprotein	360579,3
XP_029826783.1	Tyrosine protein kinase SYK	Transferase	106476,3
XP_029826115.1	tRNA dimethylallyltransferase	IPP transferase	54332,1
XP_029826317.1	Pcf11 pre-mRNA cleavage complex 2 protein (isoforms X1-X4, X8)	Glycosyltransferase	179340,6
XP_029842754.1	Nicotinate phosphoribosyltransferase (isoforms X1-X4)	NAPRTase/ Transferase/ Glycosyltransferase	65903,3
XP_029832444.1	Pleckstrin homology domain containing family M member 1	autophagy function	82719,3
XP_002414631.2	Membrane metalloendopeptidase like 1	Peptidase M13/ Protease	86449,4
XP_029846290.1	Aminopeptidase NAALADL1	Peptidase M28	82620,8
XP_002406923.2	DNAJ homolog subfamily C member 30, mitochondrial	Chaperone	23989,5
XP_029848552.1	Guanine nucleotide-binding protein 1-like	GTPase activity	64474,8
XP_002416681.2	Leukocyte elastase inhibitor	Serpin	44265,3
XP_002401213.1	SAP30-binding protein (isoforms X1-X2)	Globin	33598
XP_029840300.1	VAC14 protein homolog (X1-X2)	VAC14	84332,9
XP_029850990.1	LOW-QUALITY PROTEIN: cytochrome b-c1 complex subunit 6, mitochondrial	UQCRH/QCR6 family	11959,2
XP_002414159.2	Nonspecific lipid transfer protein	Thiolase	43901,5
XP_029851117.1	Monocarboxylate transporter-like 3 (isoforms X1-X2)	MFS transporters	57588,8
XP_029831159.1	Carnitine O-acetyltransferase	Carnitine/ Choline acetyltransferase	70307,7
XP_029843550.1	Probable chitinase 10	Glycosyl hydrolase 18	293367,3
XP_029849871.1	Anaphase-promoting complex subunit 1	APC10	205534,5
XP_029831278.1	SEC 14-like protein 2	SFH5	45888,2
XP_002407917.1	Peptidyl-prolyl cis-trans isomerase	Cyclophilin-like PPIASE	22097

XP_029836748.1	Peptidyl-prolyl cis-trans isomerase D-like (isoforms X1-X2)	Cyclophilin-like PPIASE	46851,5
XP_029840731.1	Peptidyl-prolyl cis-trans isomerase D	Cyclophilin-like PPIASE	41476,8
XP_029840732.1	Peptidyl-prolyl cis-trans isomerase 7	Cyclophilin-like PPIASE	34784,2
XP_029841004.1	Striatinin-interacting protein 1 homolog (isoforms X1-X2)	STRIP	93981,2
XP_029850594.1	52-kDa repressor of protein kinase-like inhibitor	THAP1	34782,3
XP_029822890.1	Transient receptor potential cation channel subfamily M member 7 (isoforms X1-X2)	Protein kinase	175279,5
XP_029841636.1	TBC1-like domain family member 10B	RAB	34249,2
XP_029826722.1	Lattice protein	Protein-coupled receptor 1 G	132365,8
XP_029838727.1	PTCD3 protein homolog, mitochondrial	Mitochondria-specific ribosomal protein mS39	76676,9
XP_029826091.1	Unc-104 kinesin-like protein (isoforms X1-X5)	Kinesin	202975,1

Database accession on NCBI database (https://www.ncbi.nlm.nih.gov).

S2 Fig. **Protein families identified in *Amblyomma sculptum* tick saliva.** The largest number of identified proteins belong to the cyclophilin-type PPIASE family (11.1%).

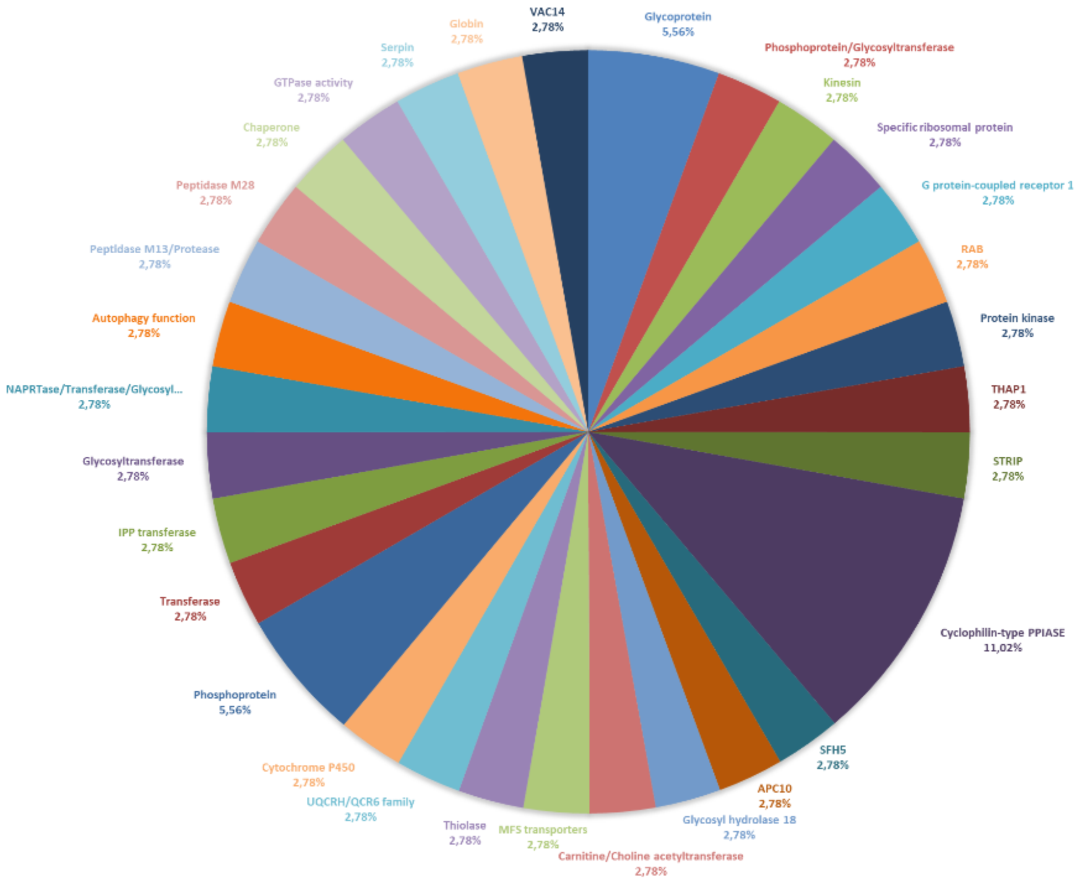

Supplement: S1 Supporting Information — (PDF) [file pone.0331779.s001.pdf]
